# Pre-Dog-Leg: A Feature Optimization Method for Visual Inertial SLAM Based on Adaptive Preconditions

**DOI:** 10.3390/s25196161

**Published:** 2025-10-04

**Authors:** Junyang Zhao, Shenhua Lv, Huixin Zhu, Yaru Li, Han Yu, Yutie Wang, Kefan Zhang

**Affiliations:** Laboratory of Intelligent Control, Rocket Force University of Engineering, Xi’an 710025, China; zhaojy8611@outlook.com (J.Z.); hgdzhx@163.com (H.Z.); 18215516817@163.com (Y.L.); 1318618716@163.com (H.Y.); wang1171506685@163.com (Y.W.); kefanzhang2024rfue@outlook.com (K.Z.)

**Keywords:** visual inertia fusion, feature optimization, preconditioner, Dog-Leg algorithm, outlier suppression, outlier suppression, trajectory accuracy

## Abstract

To address the ill-posedness of the Hessian matrix in monocular visual-inertial SLAM (Simultaneous Localization and Mapping) caused by unobservable depth of feature points, which leads to convergence difficulties and reduced robustness, this paper proposes a Pre-Dog-Leg feature optimization method based on an adaptive preconditioner. First, we propose a multi-candidate initialization method with robust characteristics. This method effectively circumvents erroneous depth initialization by introducing multiple depth assumptions and geometric consistency constraints. Second, we address the pathology of the Hessian matrix of the feature points by constructing a hybrid SPAI-Jacobi adaptive preconditioner. This preconditioner is capable of identifying matrix pathology and dynamically enabling preconditioning as a strategy. Finally, we construct a hybrid adaptive preconditioner for the traditional Dog-Leg numerical optimization method. To address the issue of degraded convergence performance when solving pathological problems, we map the pathological optimization problem from the original parameter space to a well-conditioned preconditioned space. The optimization equivalence is maintained by variable recovery. The experiments on the EuRoC dataset show that the method reduces the number of Hessian matrix conditionals by a factor of 7.9, effectively suppresses outliers, and significantly improves the overall convergence time. From the analysis of trajectory error, the absolute trajectory error is reduced by up to 16.48% relative to RVIO2 on the MH_01 sequence, 20.83% relative to VINS-mono on the MH_02 sequence, and up to 14.73% relative to VINS-mono and 34.0% relative to OpenVINS on the highly dynamic MH_05 sequence, indicating that the algorithm achieves higher localization accuracy and stronger system robustness.

## 1. Introduction

In operational scenarios involving both low-speed and high-speed motion, the combination of camera and Inertial Measurement Unit (IMU) sensors offers complementary advantages in data reliability. While IMUs suffer from long-term drift, they provide high-frequency, low-latency relative motion estimates over short durations. Conversely, visual sensors offer globally consistent observations but are prone to failure under low-texture or dynamic lighting conditions. The complementarity between visual and inertial measurements makes them particularly well-suited for sensor fusion. By jointly leveraging the strengths of both sensors, visual-inertial systems can achieve accurate and robust state estimation, even in challenging environments. Such fusion techniques are critical in domains requiring precise localization and consistent mapping, including autonomous driving, augmented reality, and drone navigation.

The core of visual inertial fusion is the state estimation problem, which essentially involves using observation data from cameras and IMUs to infer the internal state of the fusion system through probabilistic models. State estimation methods primarily rely on filtering and optimization techniques, as demonstrated in [1,2,3,4,5,6,7] filtering updates. The filtering update process reconstructs the entire covariance matrix, with its computational complexity growing quadratically with the number of state dimensions. This makes it difficult to meet the real-time requirements of high-dynamic scenes and lacks the ability for global optimization of historical data. Optimization methods, on the other hand, use incremental updates that only update the information matrix locally, leveraging the sparsity of the matrix to avoid reconstructing the covariance matrix. The monocular vision-inertial odometry system Visual-Inertial System—Monocular (VINS-mono) [8] employs a sliding window algorithm for iterative updates, enabling joint estimation of the camera and IMU. Building on this, the authors of [9,10], respectively, applied different state estimators for real-time optimization within the sliding window. However, when the number of parameters to be optimized increases, issues such as slow convergence speed and slow system response arise, which are often undesirable in practical engineering applications. Marginalization techniques in sliding window optimization effectively reduce the optimization volume. In [11,12], marginalization techniques combined with Schur complement and feature reordering are used to effectively filter out old frames. In [13], a QR decomposition marginalization technique was proposed and applied to global Bundle Adjustment (BA) optimization. The core of this method is to form marginalization constraints by projecting onto the null space of the matrix via QR decomposition. This method employs a different marginalization approach from Schur complementation and has validated the algorithm’s real-time efficiency. The real-time integration of sliding window marginalization with factor graph optimization [14,15,16] has become the primary technology for camera–IMU fusion.

In backend factor graph optimization, the complexity of the information matrix directly impacts the core performance of the optimization algorithm. For example, the loss of sparsity can lead to a significant increase in non-zero elements, thereby increasing the algorithm’s solution time and reducing system efficiency. Similarly, as the condition number of the information matrix continues to deteriorate during the solution process, it can introduce unnecessary errors and even distort the entire solution result. In SLAM, the zero space of the information matrix corresponding to the unobservable direction is the primary factor causing an increase in the condition number of the information matrix. The unobservability of the global pose leads to a zero space with four degrees of freedom in the information matrix, ultimately resulting in cumulative error growing quadratically with distance. In [17,18,19,20], the information matrix is modeled as a square root form, and the large condition number is distributed across the decomposed matrices, thereby reducing error accumulation during iterative calculations. In Robocentric Visual-Inertial Odometry 2 (RVIO2) [21], the robot’s body coordinate system is used as the reference frame to estimate the relative motion between adjacent states, and the global pose is reconstructed by accumulating relative poses. This method effectively reduces the condition number of the global pose. Additionally, for large-scale matrix solving problems, preconditioners can be introduced to accelerate algorithm convergence. In this regard, refs. [22,23,24,25] developed various preprocessing techniques, significantly increasing the solution rate of the least squares problem. Building on this, PC-SRIF [26] extracts global pose blocks and applies a preconditioning mechanism to these blocks, making Cholesky decomposition stable in single precision. Furthermore, the observability-constrained method based on the first-order estimated Jacobian (FEJ) [27,28] effectively ensures observability and prevents the deterioration of the matrix condition number. Orb-slam3 [29] and VINS-mono [8] also adopt this method to fix the linearization point of the Jacobian matrix. Additionally, the ill-posed nature of the feature point parameter information matrix significantly impacts performance. Ref. [30] leverages deep learning knowledge as prior information, effectively mitigating ill-posed information matrices. Meanwhile, ref. [31] performs visual-inertial initialization in stages, demonstrating robustness in suppressing insufficient disparity issues. Ref. [32] identifies pure rotation frames and designates them as special subframes in the backend BA, providing additional constraints. This effectively mitigates Hessian near-degeneracy caused by feature mismatches and pure rotational motion, which leads to insufficient parallax information.

**Figure 1:**
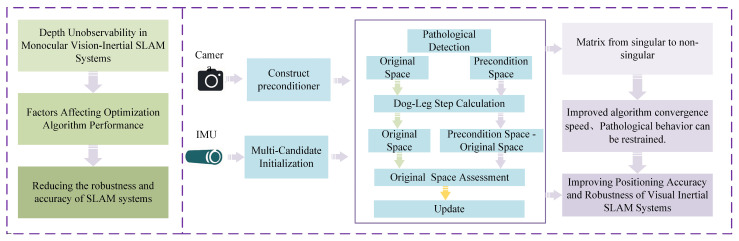
Research framework.

As shown in Table 1, fewer studies address the ill conditioning of the Hessian matrix associated with the feature point parameter. Most existing methods rely on deep prior knowledge to constrain algorithmic stability, but such approaches are only applicable in specific scenarios and do not fundamentally alleviate matrix ill-posedness at the algorithmic level. However, an optimization algorithm capable of effectively handling ill-posed matrices is indispensable for SLAM systems, particularly when external prior information or sensor assistance is lacking. This issue not only significantly reduces the efficiency of the solution but also amplifies errors during matrix inversion, affecting optimization convergence and system stability, especially in long-duration SLAM systems. To mitigate the impact of feature point information matrix ill-posedness on SLAM systems at the algorithmic level, we propose the Pre-Dog-Leg algorithm. The research framework is illustrated in Figure 1. Our main contributions are as follows:

1. We model feature-point information as Multi-State Constraint Kalman Filter (MSCKF) [33] features and propose a robust multi-candidate initializer. In addition, we introduce an adaptive SPAI-Jacobi preconditioning mechanism tailored to feature-point information: it automatically activates when the Hessian becomes ill-conditioned (large condition number) while incurring no extra overhead for well-conditioned problems.
2. To address the real-time bottleneck of Dog-Leg [34] in high-dimensional nonlinear SLAM—namely excessive iterations and poor convergence—we integrate Dog-Leg into feature-point estimation and enhance it with preconditioning: Dog-Leg updates are performed in the preconditioned parameter space, and the increments are pulled back via the inverse preconditioning operator to the original state space, suppressing pathological behavior, accelerating convergence, and improving accuracy and robustness.
3. Experiments show that the feature-point-driven preconditioner effectively reduces the Hessian condition number and markedly speeds up Dog-Leg convergence, improving overall efficiency in high-dimensional nonlinear SLAM. On the EuRoC dataset, our method achieves lower absolute trajectory error than RVIO2, VINS-Mono, and OpenVINS, demonstrating higher positioning accuracy and robustness and confirming its practical effectiveness in complex environments.

## 2. Pre-Conditioned Construction Principle

### 2.1. Analysis of the Characteristic Point Parameter Pathology Problem

In visual inertial SLAM systems, accurate estimation of feature point parameters is crucial for achieving high-precision localization and mapping. However, due to the inherent characteristics of visual observations and the high-dimensional complexity of system states, feature point parameter estimation often faces severe numerical ill-posedness. This ill-posedness not only affects the localization accuracy of the feature points themselves but also propagates through coupling relationships during the optimization process to the entire system’s state estimation, becoming a significant bottleneck constraining SLAM system performance.

**Figure 2:**
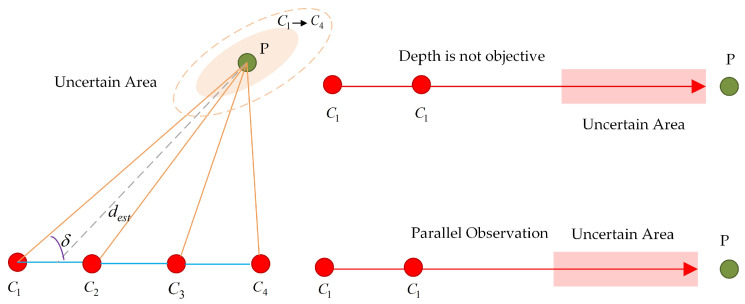
Pathological Source Analysis.

In Figure 2, $C_{i}$ denotes camera measurements at different positions. When cameras simultaneously observe feature point P, different parallaxes δ exist. When parallax δ is small, depth $d_{est}$ becomes highly sensitive to observation noise, leading to significant uncertainty regions in the depth estimates. Conversely, when parallax is large, the uncertainty regions are relatively larger (as indicated by the dashed ellipses in the figure). This uncertainty in depth estimation directly manifests in the Hessian matrix of the optimization problem: when feature point depths are difficult to observe precisely, the corresponding Hessian sub-block approaches singularity, significantly increasing the matrix’s condition number and introducing numerical ill-posedness. Similarly, in Figure 1, when the lines of sight from two observations are nearly parallel, the parallax approaches zero, making depth nearly unobservable. In this degenerate case, depth information for feature points cannot be extracted from visual measurements.

Additionally, the state vector of visual inertial SLAM typically includes parameters such as pose and velocity at consecutive time steps, as well as feature point inverse depth, resulting in a banded structure for the system’s error covariance matrix. Within the global Hessian matrix H there exist coupling blocks related to feature point parameters. Errors in these parameters propagate to the pose component via the chain rule. The ill-posed nature of the feature point Hessian directly correlates with feature parameter errors, thereby impacting the accuracy of state estimation.

Define the condition number as

(1) $$k(H)=\frac{\lambda_{\max}(H_{f})}{\lambda_{\min}(H_{f})}$$

$H_{f}$ is the Hessian matrix of the feature points, and in monocular vision inertial fusion slam, often the depth information is unobservable. When the initial difference between the depth parameter $\rho =1/d_{\mathrm{est}}$ (where $d_{\mathrm{est}}$ is the metric depth in meters) number and the angle parameter θ,φ (pitch angle and yaw angle) is too large, it will lead to an exponential increase in the value of k(H).

Therefore, the estimated value Δx for the feature point parameter should satisfy the following requirements:

(2) $$\frac{∥\Delta x-\Delta x^{*}∥}{∥\Delta x^{*}∥}\leq k(H)\frac{∥\sigma g∥}{∥g∥}+O(\varepsilon^{2})$$

where Δx denotes the solution obtained by computation, $\Delta x^{*}$ is the real solution, and σg and g denote the perturbation of the gradient and the gradient vector, respectively. It can be seen from Equation (2) that even a small relative error in the gradient is infinitely amplified by the condition number k(H). Similarly, the condition number also affects the convergence speed of the algorithm:

(3) $$\begin{array}{l} R=\frac{k(H)-1}{k(H)-1}\approx 1-\frac{2}{k(H)}\\ N=\frac{k(H)}{2}\ln(\frac{1}{\varepsilon }) \end{array}$$

R and N denote the rate of convergence and the number of iterations, respectively. As seen from Equation (3), the larger the number of conditions, the slower the convergence, and the more iterations are required. The fundamental purpose of preconditioning is to speed up the convergence of the algorithm by transforming the original problem to make it easier to solve numerically.

### 2.2. Methods of Constructing Preconditioned Matrices

In inertial vision SLAM, preconditioning mechanisms are used to improve the numerical conditions of the optimization problem. It is usually necessary to solve the least squares problem with a regular equation of the form:

(4) H⋅Δx=b

where $H=J^{T}J$ is the Jacobi matrix, Δx is the state increment, and b is the residual vector.

The core idea of the preconditioning technique is that the original problem is transformed into an equivalent problem with a better condition number by introducing a preconditioned matrix P. Ideally, the preconditioned matrix is an approximation of the original matrix H. The preconditioning brings the matrix close to the unit matrix:

(5) $$\hat{H}=P\cdot H\approx E$$

In order to achieve an improvement in the number of conditions, pre-conditioning techniques are usually constructed in three ways:

Left pre-conditions:

(6) P⋅HΔx=P⋅b

Right pre-condition:

(7) H⋅PΔx=b,x=Py

The splitting preconditioner, in terms of the incomplete Cholesky of P, is set, and then the splitting preconditioner is as follows:

(8) $$L^{-1}{\mathrm{HL}}^{-T}\cdot y=L^{-1}b,x=L^{-T}y$$

where P is the preconditioning matrix. A good preconditioned matrix PH should satisfy the following: PH is benign with a good condition number; the preconditioned matrix PH should be benign, with a good condition number. The eigenvalues of H should be clustered and far from zero. Additionally, the preconditioned matrix P should be computationally inexpensive to construct.

However, traditional preconditioner construction methods face various degrees of limitations when addressing the feature point optimization problem in visual inertial SLAM. The diagonal preconditioner Jacobi approach only scales the diagonal elements, ignoring the strong coupling between the inverse depth and angle parameters. The fixed sparse mode of the SPAI approach is difficult to adapt to the dynamic changes in the Hessian matrix of the feature points. To address these issues, we combine the SPAI [35] and Jacobi [36] approaches to construct the preconditioner. We also deeply integrate the Dog-Leg optimization process. On this basis, an algorithm of Pre-Dog-Leg is proposed specifically for the feature point parameter pathology problem.

## 3. Pre-Dog-Leg Based Feature Point Optimization Approach

### 3.1. Multi-Candidate Initialization Strategy

Traditional Cartesian coordinates are prone to optimization difficulties due to scale uncertainty. Inverse depth parameterization expresses three-dimensional points through direction vectors and inverse depth, parameterizing feature points as pitch angle (ϕ), yaw angle (ψ), and inverse depth (ρ=1/d), based on the feature point measurement formula [37]; the following can be expressed as

(9) $$P_{c}=\frac{1}{\rho }\left[\begin{array}{c} \sin\psi \cos\phi \\ \sin\phi \\ \cos\psi \cos\phi \end{array}\right]$$

Due to the unobservability of depth information, the initial value of depth information directly determines the initial performance of the optimization algorithm and directly affects the accuracy of the 3D map. We use the triangulation formula to infer depth information from parallax. Normalizing the focal length to f, with the baseline length denoted as b and the pixel parallax as $\delta =z_{1}-z_{2}$, the initial depth information can be expressed as

(10) $$d_{\mathrm{est}}=\frac{b}{\delta +\epsilon }$$

where $z_{1}$ is the first frame image coordinates, $z_{2}$ is the current frame image coordinates, and ϵ is the regularization term;

Using Formula (6), we generate a candidate set $\left\{0.5d_{est},d_{est},2.0d_{est}\right\}$ for depth. The generated depth candidate set covers a logarithmic spatial interval $\left\{\ln(0.5d_{est}),\ln(d_{est}),\ln(2.0d_{est})\right\}$ around the estimated depth. For depth information, we generate a candidate set of depth information that transforms single-point initialization into an initialization method based on geometric constraints in parameter space search, thereby avoiding the problem of high condition numbers in the Hessian matrix caused by incorrect initialization of depth information. Similarly, the initialization of the angle is performed by initializing the direction angle using pixel coordinates $\left\{u,v,1\right\}$:

(11) $$\left\{\begin{array}{l} \phi_{\mathrm{init}\;}=\arctan\left(\frac{v}{\sqrt{u^{2}+1}}\right)\\ \psi_{\mathrm{init}\;}=\arctan\left(\frac{u}{\sqrt{u^{2}+v^{2}+1}}\right) \end{array}\right.$$

where $\phi_{\mathrm{init}\;}$ is the pitch angle, $\psi_{\mathrm{init}\;}$ is the yaw angle, v is the horizontal coordinate of the image plane, and u is the vertical coordinate of the image plane.

Due to the low complexity of our deep candidate set, no additional costs are incurred during the actual initialization process. However, in practical applications, correct initialization alone cannot fully improve the condition number of the Hessian matrix and the stability of the algorithm. It must be used in conjunction with the preconditioner we have constructed.

### 3.2. Combination Preconditioner Design

The purpose of our preconditioning is to explicitly construct a preconditioned matrix P such that the condition number of the Hessian matrix after a preconditioned change is reduced:

(12) P⋅HΔx=P⋅b

The usual construction $\hat{H}=\mathrm{PH}\approx I$, which transforms the problem into a quadratic optimization problem without coupling in the ideal case, can speed up the iteration rate and stability of the algorithm. However, we consider that such a construction will lose the positive definiteness of the Hessian matrix, so we perform a symmetric construction of the Hessian matrix:

(13) $$\hat{H}=P^{T}\mathrm{HP}\approx I$$

where P is the preconditioning matrix, H is the global Hessian matrix, and I is the unit matrix.

Due to the dimensional differences between depth information and angle information, simple scaling and balancing of scales cannot reduce the condition number of the Hessian matrix. The real issue lies in the nonlinear geometric coupling between parameters. We approximate the inverse of the Hessian matrix based on inverse depth information using SPAI, but SPAI cannot resolve the fundamental matrix ill-conditioned problem. Therefore, we combine the Jacobi method with column norm averaging of the matrix after SPAI to further balance the numerical magnitude.

SPAI construction method:

(14) $$P_{\mathrm{SPAI}}=\left\{\begin{array}{cc} \frac{1}{\max\left(H_{\mathrm{ii}},\epsilon \right)}, & i=j\\ -\beta \cdot \frac{H_{\mathrm{ij}}}{\max\left(H_{\mathrm{ii}},\epsilon \right)}, & i\neq j\\ 0, & \mathrm{else} \end{array}\right.$$

where β is the regularization parameter, i and j are the row and column index values, $H_{\mathrm{ij}}$ is the main diagonal element, and $H_{\mathrm{ij}}$ is the non-diagonal element.

Structure $P_{SPAI}$, the reciprocal of the diagonal elements of the Hessian matrix, is taken for the diagonal elements, and a regularization term ϵ is introduced to prevent division by zero. For the off-diagonal elements, filtering is performed based on the maximum diagonal element of H. When $\;\left|H_{\mathrm{ij}}\right|>\alpha \cdot \max_{\mathrm{diag}\;}$ non-zero terms are added, β is used for the weak coupling correction of the off-diagonal elements.

Jacobi preconditioner:

(15) $$N=\mathrm{diag}\left({\left\|{\left(P_{\mathrm{SPAI}\;}^{T}H\right)}_{i}\right\|}_{2}^{-1}\right)$$

For the Jacobi preconditioner, we introduce $P_{\mathrm{SPAI}\;}^{T}H$ to scale the norm of this matrix. The core is to take the inverse of the L2 norm of each row of the transformation matrix processed by $P_{\mathrm{SPAI}\;}^{T}H$ to construct a diagonal scaling matrix.

Finally, perform SPAI-Jacobi combination preconditioning to construct preconditioning sub-problems:

(16) $$P={P^{T}}_{\mathrm{SPAI}}\cdot N$$

The inverse depth preconditioner construction process is presented in Algorithm 1. The SPAI preconditioner constructs an approximate inverse matrix, retaining only the important parameter relationships, and directly approximates $H^{-1}$. The Jacobi preconditioner eliminates the remaining imbalance after SPAI. The combined preconditioner uses a dual strategy of sparse approximation and non-uniform scaling to significantly reduce the condition number of the Hessian, making it particularly suitable for problems with large differences in the scale of direction angles and depth. However, in practical problems, the condition number of the feature point Hessian matrix does not continuously increase. When there is a smaller disparity range and more observations, the Hessian matrix is well-conditioned and meets optimization requirements. Therefore, we set a threshold for the preconditioner, which is triggered only when the matrix is ill-conditioned to reduce its condition number.
**Algorithm 1:** Build Inverse DepthPreconditioner**Input:** Hessian matrix $H\in R^{3\times 3}$ **Output:** P_combined $P\in R^{3\times 3}$ **# 1. Initialization**
Set preconditioner_ready = false Set P_combined = Identity **# 2. Check Conditioning** If k(H)≤1000: return $I_{3}$ where $K(H)=\delta_{\max}/\delta_{\min}$
**# 3. Build SPAI Preconditioner** Diagonal: $P_{SPAI}(i,i)=1/\max\left(H_{ii},\epsilon \right)$ Off-diagonal: $-\beta \cdot H_{ij}/\max\left(H_{ii},\epsilon \right)$ and i ≠ j where α β=0.05, $\varepsilon =10^{-12}$
**# 4. Construct Jacobi Preconditioner** $\;\;N=P_{\mathrm{SPAI}}^{T}\times H$
For row = 0 to 2: $\;\;N=\mathrm{diag}\left({\left\|{\left(P_{\mathrm{SPAI}\;}^{T}H\right)}_{i}\right\|}_{2}^{-1}\right)$
End for **# 5. Combine Preconditioners**
P_combined = P_spai × N Return P_combined


### 3.3. Pre-Dog-Leg Algorithm

In the SIRF based on QR decomposition, the state variables are continuously updated through iterative QR decomposition, ensuring both accuracy and efficiency. However, when solving the least squares problem of the cost function, methods such as Levenberg–Marquardt (LM), QR decomposition, Singular Value Decomposition (SVD) decomposition, and Dog-Leg are all affected by strong coupling and scale differences between parameters. The convergence speed and numerical stability are closely related to the ill-conditioned nature of the matrix. In the estimation of inverse depth parameters, rounding errors often propagate through the chain rule to pose information, and the accuracy of the estimation directly determines the accuracy of localization and 3D reconstruction. In contrast, iterative algorithms like Dog-Leg exhibit higher accuracy and efficiency, but they may fail to converge or converge slowly when faced with matrix ill-posedness. Therefore, we improved Dog-Leg based on the designed preconditioner, performing Dog-Leg steps in the parameter space after preconditioning transformation, effectively addressing the issues of scale differences and strong coupling between parameters, and proving mathematical equivalence.

For each measurement point I, the residual is defined as

(17) $$r_{i}(x)=z_{i}-\pi (h_{i})=\left[\begin{array}{l} u_{i}-\frac{h_{i,x}}{h_{i,z}}\\ v_{i}-\frac{h_{i,y}}{h_{i,z}} \end{array}\right]$$

Among them, $z_{i}={\left[u_{i},v_{i}\right]}^{T}$ is the observation point on the normalized plane, $h_{i}=R_{c}^{i}e_{inv}+\rho t_{c}^{i}$ is the coordinate of the point in the camera coordinate system, and $e_{inv}={\left[\cos\phi \sin\psi ,\sin\phi ,\cos\phi \cos\psi \right]}^{T}$ is the unit direction vector of the inverse depth parameterization.

The cost function and Jacobian matrix can be written as

(18) $$\begin{array}{l} C=\frac{1}{2}\sum_{i=1}^{N}{\left\|r_{i}(x)\right\|}^{2}=\frac{1}{2}r{\left(x\right)}^{⊤}r(x)\\ H_{i}=-H_{{\mathrm{pro}}_{j,i}}\left[R_{c_{i}},J_{\mathrm{ang}},t_{c_{i}}\right] \end{array}$$

Among them, $r(x)={\left[r_{1}^{T},r_{2}^{T},\ldots ,r_{N}^{T}\right]}^{T}$ is the vector of all residual stacks, and x=[ϕ,ψ,ρ] is the optimization parameter. $H_{{\mathrm{pro}}_{j,i}}$ and $J_{\mathrm{ang}}$ are the projection Jacobian matrix and angle Jacobian matrix, respectively. The Hessian matrix and the original gradient g can be expressed as

(19) $$\begin{array}{l} H=J{\left(x\right)}^{T}J(x)\\ g=J{\left(x\right)}^{T}r(x) \end{array}$$

Based on the above, we introduce the preconditioner P and redefine the effective gradient g and Hessian matrix in the preconditioned space:

(20) $$\begin{array}{l} g_{\mathrm{eff}}=P^{T}g\\ H_{\mathrm{eff}}=P^{T}\mathrm{HP} \end{array}$$

In the precondition space, the steepest descent direction can be expressed as

(21) $$\alpha_{\mathrm{eff}}=\frac{g_{\mathrm{eff}}^{T}g_{\mathrm{eff}}}{g_{\mathrm{eff}}^{T}H_{\mathrm{eff}}g_{\mathrm{eff}}}$$

where $\alpha_{\mathrm{eff}}$ is the effective step size in the preconditioned space, $g_{\mathrm{eff}}$ is the gradient in the preconditioned space, and $H_{\mathrm{eff}}$ is the Hessian matrix in the preconditioned space;

The steepest descent step size $p_{\mathrm{sd},\mathrm{eff}}$ based on the preconditioner is

(22) $$p_{\mathrm{sd},\mathrm{eff}}=-\alpha_{\mathrm{eff}}g_{\mathrm{eff}}$$

In the preconditioned space, the Gauss–Newton direction solution can be transformed into

(23) $$H_{\mathrm{eff}}p_{\mathrm{gn},\mathrm{eff}}=-g_{\mathrm{eff}}$$

Using LDLT decomposition, we obtain

(24) $$p_{\mathrm{gn},\mathrm{eff}}=-H_{\mathrm{eff}}^{-1}g_{\mathrm{eff}}$$

In the precondition space, let the trust region radius be Δ; then, the Dog-Leg interpolation cases are as follows:

(25) $$\left\{\begin{array}{cc} p_{\mathrm{dl},\mathrm{eff}}=p_{\mathrm{gn},\mathrm{eff}}, & \left\|p_{\mathrm{gn}}\right\|\leq \Delta \\ p_{\mathrm{dl},\mathrm{eff}}=\frac{\Delta }{\left\|p_{\mathrm{sd},\mathrm{eff}}\right\|}p_{\mathrm{sd},\mathrm{eff}}, & L_{\mathrm{gn}}>\Delta ,\;\alpha_{\mathrm{eff}}\left\|p_{\mathrm{sd}}\right\|\geq \Delta \\ p_{\mathrm{dl}}=a+\beta^{*}c, & \mathrm{else} \end{array}\right.$$

where $p_{\mathrm{dl},\mathrm{eff}}$ is the preconditioned spatial step, $L_{\mathrm{gn}}$ is the quadratic model predicted descent in the Gaussian–Newtonian direction, $p_{\mathrm{sd}}$ is the steepest descent step in the original space, $p_{\mathrm{gn}}$ is the Gaussian–Newtonian step in the original space, and $\beta^{*}$ is the weight.

Equation (25) implements a dual-space optimization strategy, where the feasibility of the step size is evaluated in the original parameter space, while the actual optimization iteration occurs in the preconditioned space. Specifically, when $\left\|p_{\mathrm{gn}}\right\|\leq \Delta$, the Gauss–Newton step size is within the trust region, and direct use yields the fastest convergence. When $L_{\mathrm{gn}}>\Delta ,\alpha_{\mathrm{eff}}\left\|p_{\mathrm{sd}}\right\|\geq \Delta$, the optimal step size for the steepest descent exceeds the trust region, resulting in linear convergence with high stability but reduced convergence speed. The third case represents the essence of the Dog-Leg algorithm: constructing a “dog-leg” path in the preconditioned space, starting from the steepest descent step $p_{\mathrm{sd},\mathrm{eff}}$ and adjusting direction c through parameter $\beta^{*}$, ultimately reaching the optimal point on the trust region boundary.

Let $a=\alpha_{\mathrm{eff}}p_{\mathrm{sd},\mathrm{eff}},c=b-a$, and solve the quadratic equation:

(26) $$\begin{array}{l} \|a+\beta c{\|}^{2}=\Delta^{2}\\ \|a{\|}^{2}+2\beta a^{T}c+\beta^{2}\|c{\|}^{2}=\Delta^{2} \end{array}$$

When the discriminant is $\Delta_{\mathrm{disc}\;}={\left(a^{T}c\right)}^{2}+\|c{\|}^{2}\left(\Delta^{2}-\|c{\|}^{2}\right)$, we can solve for

(27) $$\beta =\frac{\sqrt{\Delta_{\mathrm{disc}}}-a^{T}c}{\|c{\|}^{2}}$$

Dog-Leg stride length:

(28) $$p_{\mathrm{dl},\mathrm{eff}}=a+\beta c$$

After performing the transformation in the precondition space, substitute the solution back into the original space:

(29) $$p_{\mathrm{dl}}=Pp_{\mathrm{dl},\mathrm{eff}}$$

For updates to the region of trust, the actual cost $\Delta f_{\mathrm{actual}\;}$ is represented by the decrease in the objective function after moving the current parameter θ to $\theta +p_{\mathrm{dl}}$:

(30) $$\Delta f_{\mathrm{actual}\;}=f(\theta )-f\left(\theta +p_{\mathrm{dl}}\right)=\frac{1}{2}\|r(\theta ){\|}^{2}-\frac{1}{2}{\left\|r\left(\theta +p_{\mathrm{dl}}\right)\right\|}^{2}$$

To ensure scale consistency, we convert the prediction cost into the original space for calculation:

(31) $$\Delta f_{\mathrm{pred}}=-g^{T}p_{\mathrm{dl}}-\frac{1}{2}p_{\mathrm{dl}}^{T}Hp_{\mathrm{dl}}$$

Among them, $p_{\mathrm{dl}}=P{\tilde{p}}_{\mathrm{dl},\mathrm{eff}}$ is the stride in the preconditioned space, $g=P^{T}{\tilde{g}}_{\mathrm{eff}}$ is the gradient in the preconditioned space, and ${H=P}^{T}{\tilde{H}}_{\mathrm{eff}}P$ is the Hessian in the preconditioned space.

The ratio of the confidence domain can be expressed as

(32) $$\rho =\frac{\Delta f_{\mathrm{actual}\;}}{\Delta f_{\mathrm{pred}\;}}$$

**Figure 3:**
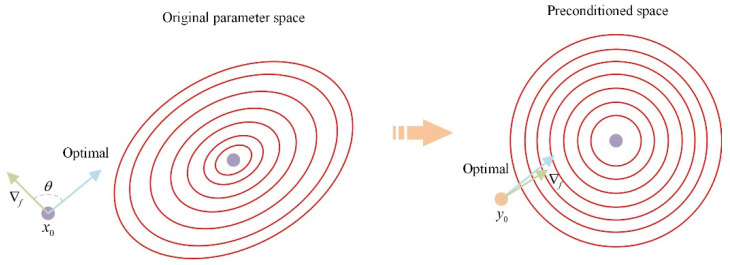
Changes in the preconditioned space trust region.

As shown in Figure 3, in the original parameter space, due to strong coupling between parameters and different numerical scales, the objective function exhibits elliptical contour distributions, resulting in significant deviations between the gradient direction and the optimal path; this leads to slow convergence and a tortuous path. By combining the SPAI preconditioner and the improved Jacobi preconditioner, the elliptical contours are adjusted to an approximately circular distribution, ensuring that the gradient direction aligns closely with the optimal direction, significantly improving convergence speed and stability. The Pre-Dog-Leg algorithm based on preconditions is shown below (Algorithm 2):
**Algorithm 2:** Pre-Dog-Leg algorithm based on preconditions$\text{Input:}\;\mathrm{Initial}\;\mathrm{parameters}\;x_{0}$, objective f(x), Jacobian J(x) $\text{Output:}\;\mathrm{Optimized}\;\mathrm{parameters}\;x^{*}$, convergence flag **converged** **#1. Multi-candidate initialization:** $\mathrm{baseline}\;\mathrm{and}\;\mathrm{parallax}\;\mathrm{generate}\;\mathrm{depth}\;\mathrm{candidate}\;\mathrm{set}\text{:}\;\left\{0.5d_{est},d_{est},2.0d_{est}\right\}$ **#2. Trust region initialization and main optimization loop:**    radius = 0.05 (initial trust region radius)    iteration = 0 $\;\;\;\;\;x=x_{0}$    while iteration < 80 and converged **#3. Preconditioned space transformation:** $\;\;\;\;\mathrm{if}\;P\neq I\;g_{e\mathrm{ff}}=P^{T}g$$,\;H_{\mathrm{eff}}=P^{T}\mathrm{HP}$# Preconditioned gradient $\;\;\;\;\mathrm{else}\;g_{\mathrm{eff}}=g$$,\;H_{\mathrm{eff}}=H$ # Preconditioned Hessian **#4. Dog-Leg step calculation:** $\mathrm{Cauchy}\;\mathrm{step}\;(\mathrm{steepest}\;\mathrm{descent})\text{:}\;p_{\mathrm{sd},\mathrm{eff}}=-\alpha_{\mathrm{eff}}g_{\mathrm{eff}}$ $\mathrm{Gauss}\text{-}\mathrm{Newton}\;\mathrm{step}\text{:}\;p_{\mathrm{gn},\mathrm{eff}}=-H_{\mathrm{eff}}^{-1}g_{\mathrm{eff}}$

Else: Dog-Leg combination step calculation **#5. Transform back to original parameter space:** $\;\;\;\;\mathrm{if}\;P\;\text{≠}\;I:\;p_{\mathrm{dl}}=Pp_{\mathrm{dl},\mathrm{eff}}$    # Transform back from preconditioned space $\;\;\;\;\mathrm{else}\;p_{\mathrm{dl}}=p_{\mathrm{dl},\mathrm{eff}}$                # Keep original space step **#6. Model prediction reduction** $\;\;\;\;\Delta f_{\mathrm{actual}\;}=f(\theta )-f\left(\theta +p_{\mathrm{dl}}\right)=\frac{1}{2}\|r(\theta ){\|}^{2}-\frac{1}{2}{\left\|r\left(\theta +p_{\mathrm{dl}}\right)\right\|}^{2}$ **#7. Status update:** if a > 0.05: x = x_new                      # Accept update Hessian = J(x)ᵀ × J(x)    # Update Hessian **#8. Radius Updates** if a > 0.9            radius = min(2.0, radius × 1.8) else if a < 0.05 radius = max(10^−6^ radius × 0.3)

## 4. Experiment

To validate the effectiveness of the proposed algorithm, simulation experiments were conducted using the publicly available EuRoC dataset, and evaluations were performed using the EVO [38] tool. The ground truth for the EuRoC dataset was provided by high-precision laser tracking scanners and motion capture systems, both of which have millimeter-level accuracy. The EuRoC dataset is categorized into three difficulty levels—hard, medium, and easy—based on data acquisition complexity. Data is collected from these three scenarios by controlling the vehicle’s speed and environmental conditions. Depending on the characteristics and difficulty of different image sequences, they can be summarized as shown in Table 2.

To better validate the robustness of the proposed algorithm, we selected the MH dataset, which features significant changes in lighting, easily blurred images, and complex trajectories, for testing. The experiments can be divided into two parts: the first part involves using the MH_01 dataset to validate the performance of the proposed preconditioner and the stability and convergence speed of the improved algorithm; the second part involves using the MH Factory dataset to validate the improvement of the improved algorithm in terms of localization accuracy and navigation performance.

### 4.1. Pre-Dog-Leg Algorithm Condition Number Verification

**Figure 4:**
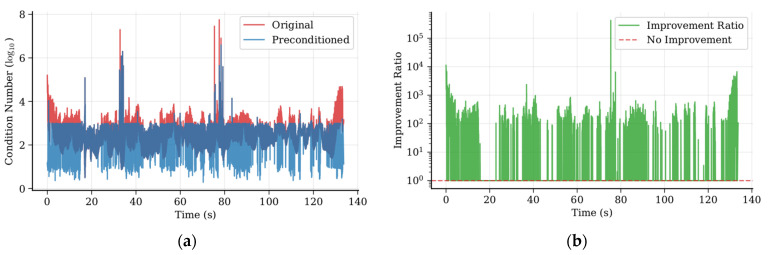

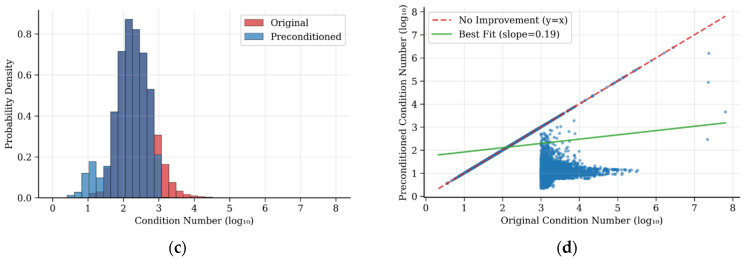
Verification of the effect of the preconditioner. (**a**) Condition number comparison; (**b**) condition number improvement factor; (**c**) condition number distribution; (**d**) precondition effectiveness.

To accurately verify the improvement in condition number, although we only applied the preconditioner to singular matrices with condition numbers greater than 10^3^, we conducted the evaluation based on the entire dataset. Figure 4a illustrates the temporal variation characteristics of the condition number of the Hessian matrix at feature points in the MH_01 sequence. The red curve represents the condition number in the original Dog-Leg algorithm, exhibiting significant fluctuations and extreme values, with values ranging from approximately 10^2^ to 10^8^, peaking near t = 30 s, t = 78 s, corresponding to visually degraded scenes. Statistical features show an average value of 2.7 × 10^3^. The blue curve indicates the effect after using the improved combined preconditioner, with the condition number effectively controlled within the range of 10 to 10^3^, the fluctuation amplitude significantly reduced, and the average value decreased to 3.4 × 10^2^. Figure 4b quantifies the improvement effect of the preconditioner, defined as the improvement multiple. The average improvement factor is approximately 7.9 times, and the improvement factor shows a positive correlation with the original condition number, performing better under numerically ill-posed conditions.

Figure 4c reveals the systematic improvement effect of the preconditioner through probability distribution analysis. The original distribution (red) exhibits typical right-skewed distribution characteristics, with a skewness of 0.655 and a kurtosis of 1.987, indicating a pronounced heavy-tailed feature. The distribution after preconditioning (blue) approximates a normal distribution, with values concentrated within the range of log_10_(1) to log_10_(3); the skewness is significantly reduced to −0.685, indicating a concentration toward the minimum value, with a kurtosis of 0.873, effectively suppressing the occurrence of extreme value pathologies. Figure 4d validates the overall effectiveness of the preconditioner through regression analysis. The scatter plot shows that the slope of the best-fit line is 0.19, indicating that the condition number after preconditioning is approximately 19% of the original value, achieving approximately 81% condition number reduction. The correlation coefficient R^2^ = 0.67 indicates that the regression model has good explanatory power, with the majority of data points located below the y = x baseline, intuitively proving the general effectiveness of the preconditioner. Notably, the preconditioner demonstrates stronger improvement capabilities in high condition number regions (log_10_ > 3), exhibiting nonlinear improvement characteristics, which aligns with the theoretical expectation that the preconditioner plays a greater role in numerically ill-posed scenarios.

Based on the above observations, the condition number does not remain in a pathological state continuously but peaks at specific moments. This observation prompted us to design an adaptive triggering mechanism rather than keep the preconditioner permanently enabled. Statistical analysis revealed that when the condition number exceeds 10^3^, the algorithm’s convergence performance deteriorates significantly. Therefore, we set 10^3^ as the activation threshold for the preconditioner. To further validate this finding, we also conducted the sensitivity tests described in Section 4.3.

**Figure 5:**
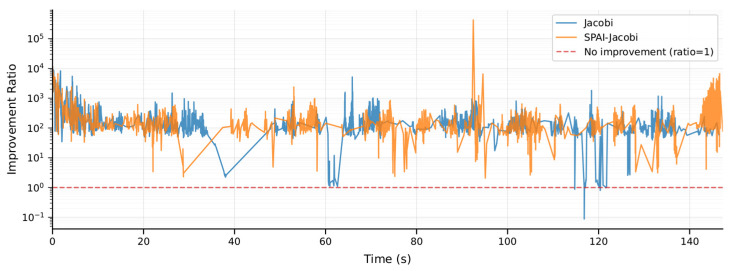
Comparison of SPAI-Jacobi and Jacobi improvement rates.

Compared to the performance of Jacobi preconditioning, we also conducted a detailed comparison between the improved SPAI-Jacobi preconditioning and the traditional Jacobi preconditioning method on the MH_01 dataset. The corresponding experimental curves are shown in Figure 5, where the horizontal axis represents time and the vertical axis represents the condition number improvement factor. The orange curve denotes the SPAI-Jacobi method, while the blue curve denotes the Jacobi method. The results reveal that SPAI-Jacobi preprocessing significantly outperforms traditional Jacobi in enhancing convergence performance. The improvement factor curve for SPAI-Jacobi consistently exceeds that of Jacobi throughout most of the timeframe and remains above 1 almost continuously. In contrast, the Jacobi method’s improvement factor occasionally approaches 1 or even drops near 1. Quantitative statistics indicate that the Jacobi method achieves an average improvement in the condition number of approximately 1.4 times, whereas SPAI-Jacobi achieves an average improvement of approximately 7.9 times, substantially outperforming the Jacobi method. Furthermore, we compared the impact of both methods on trajectory accuracy. On the MH_01 dataset, the absolute trajectory error with Jacobi preprocessing was 0.168 m, whereas the error with SPAI-Jacobi preprocessing decreased to 0.147 m. This lower trajectory error further validates the effectiveness of our method.

**Figure 6:**
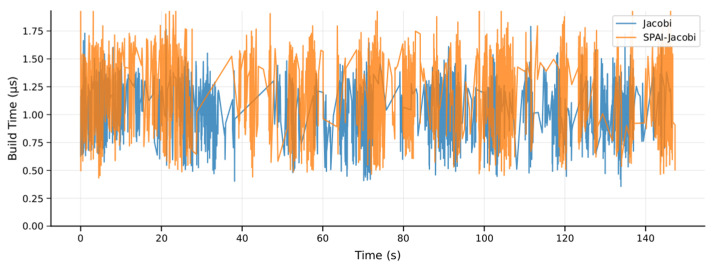
SPAI-Jacobi and Jacobi build time visualization.

**Figure 7:**
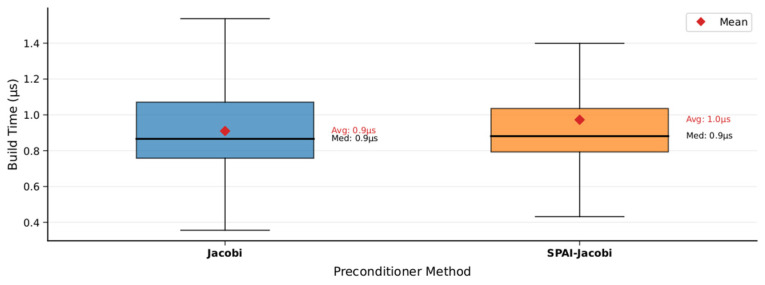
SPAI-Jacobi and Jacobi build time comparison.

In terms of preprocessor construction time, the SPAI-Jacobi method also exhibits acceptable overhead. As shown in Figure 6 and Figure 7, the SPAI-Jacobi method incurs slightly longer times during the preprocessing construction phase. Its average construction time is 0.97 μs, representing an increase of approximately 10.4% compared to the Jacobi method’s 0.88 μs. However, as is evident from the box plot in Figure 5, the majority of the distribution ranges for the construction times of both methods overlap. The SPAI-Jacobi method exhibits only a very limited additional overhead relative to the Jacobi method. Overall, the SPAI-Jacobi preprocessing achieves a significant improvement in convergence performance at a minimal cost in construction time, representing an effective enhancement over the traditional Jacobi preprocessing.

**Figure 8:**
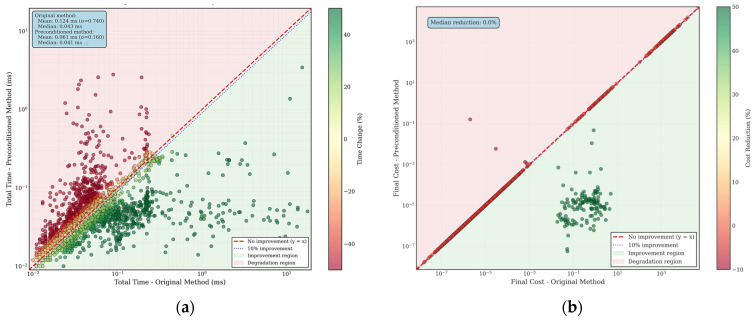
Algorithm convergence performance verification. (**a**) Time performance comparison for high condition number cases in logarithmic scale analysis; (**b**) Final cost function comparison for high condition number cases in logarithmic scale analysis.

Finally, to validate the improved Dog-Leg algorithm’s enhanced overall optimization convergence performance, we conducted a comprehensive comparison with the original Dog-Leg algorithm. Considering the visualization of data points, we randomly selected 4000 groups from samples with condition numbers exceeding 1000, ensuring each sample had an equal probability of being chosen. These high-condition-number cases better demonstrate the value of the algorithmic improvements. Figure 8a demonstrates the significant improvement in the performance of the Dog-Leg algorithm with the preconditioner. In the time performance comparison plot, the color of the data points indicates the percentage change in time, with green indicating that the preconditioned method is faster and red indicating that the preconditioned method is slower. The vast majority of the data points are located in the green improvement region below the y = x baseline, indicating a general improvement in algorithm efficiency. Specifically, the average computation time of the original algorithm is 0.124 ms, while the improved algorithm takes only 0.061 ms, a time performance improvement of about 50.8%. Notably, the medians of the original algorithms are closer (0.043 ms and 0.041 ms), which suggests that our algorithm is more stable and robust in dealing with outlier problems with high condition numbers. Figure 8b is final cost function comparison plot further validates the effectiveness of the algorithm. The high concentration of data points on the y = x diagonal and the median reduction rate of 0.0% fully demonstrate the excellent performance of the improved algorithm in maintaining the accuracy of the solution, and the preconditioning transformations do not introduce additional numerical bias. More importantly, the few data points located below the diagonal indicate that the original algorithm finds it difficult to converge to the optimal solution for some extreme pathological cases, while our preconditioning method successfully reduces the final cost function value, helping the algorithm to find a more optimal solution and, thus, improving the overall solution accuracy.

To further demonstrate the reliability of the Pre-Dog-Leg method, we will next conduct a performance comparison across the entire MH_01 and MH_05 sequences.

### 4.2. Pre-Dog-Leg Convergence Performance Experiment

In this section, we evaluate the performance of four nonlinear optimization methods on the MH01 sequence from the EuRoC dataset and the dynamic sequence MH05: the Levenberg–Marquardt (LM) algorithm, the Gauss–Newton (GN) algorithm, the Dog-Leg Trust Region algorithm, and the Pre-Dog-Leg algorithm. We integrated all four methods into our backend for testing and employed identical convergence criteria to ensure fair comparison. Key metrics evaluated include computational efficiency, trajectory accuracy, and resource consumption, with quantitative results summarized in the table below.

Table 3 illustrates the statistical characteristics of convergence time distributions for four optimization methods on the MH01 dataset. It is evident that the LM and Pre-Dog-Leg methods exhibit the fastest convergence rates, with the shortest average and median computation times. The Pre-Dog-Leg method achieves an average convergence time of approximately 0.071 ms, representing improvements of about 34.7% and 23.7% over the GN and Dog-Leg methods, respectively. Additionally, Pre-Dog-Leg exhibits higher computational stability, as evidenced by its smaller standard deviation of 0.251 ms, whereas GN and Dog-Leg demonstrate larger standard deviations and greater instability.

**Figure 9:**
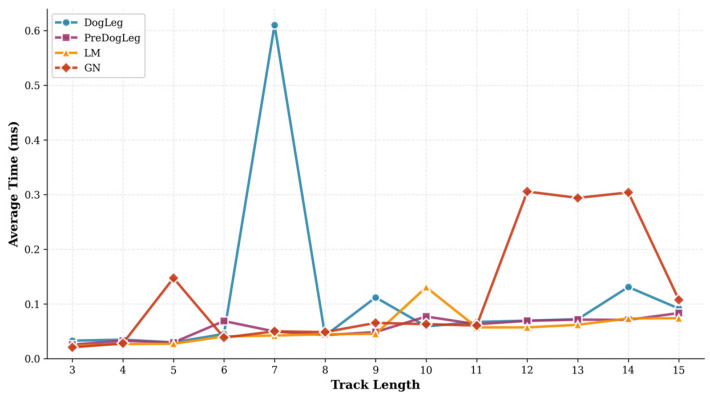
Average convergence time for different trajectory lengths (MH_01).

Figure 9 illustrates the trend of average computation time as the trajectory length varies. It is evident that the purple curve representing the Pre-Dog-Leg method consistently maintains low computational time across different trajectory lengths, showing only a slight increase as trajectories lengthen—comparable to the orange LM algorithm. The red curve representing the GN method shows a gradual increase in computational time with trajectory length, exhibiting a significant surge at longer lengths—particularly evident between lengths 11 and 15. The blue curve for the Dog-Leg method, however, displays noticeable fluctuations between medium-length trajectories 6 and 8, indicating instability when tracking shorter sequences. Overall, Pre-Dog-Leg maintains stability and low computational cost as the problem scale increases, while the computation time of GN deteriorates with scale. Dog-Leg may encounter convergence difficulties at certain medium scales, leading to sudden spikes in computational time.

**Figure 10:**
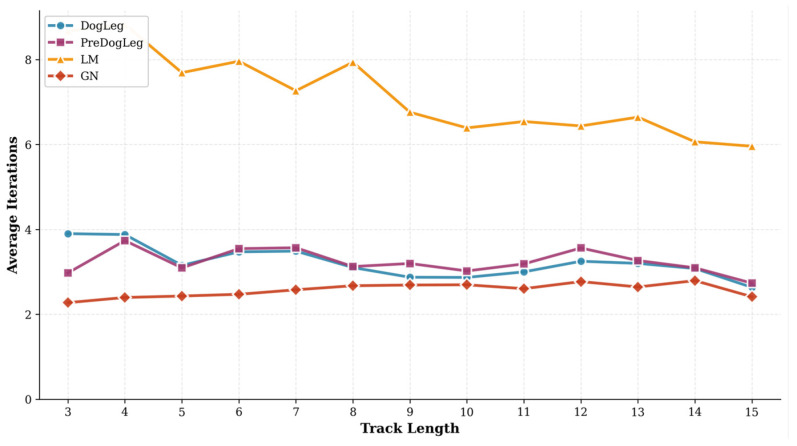
Iteration counts for different trajectory lengths (MH_01).

Furthermore, we compared the number of iterations across different trajectory lengths in Figure 10. This figure illustrates how the average number of iterations varies with trajectory length. The GN method requires the fewest iterations to converge, with only a slight increase in iterations, even as trajectories grow longer. However, the lower iteration count of GN does not translate to an overall time advantage, likely due to its larger step size per iteration and higher computational overhead per step. In contrast, the LM method requires the most iterations, peaking at short trajectory lengths but decreasing as the trajectory length increases. This suggests LM converges stably through small step sizes. Despite its higher iteration count, the reduced computational load per step keeps its total runtime low. The Pre-Dog-Leg and Dog-Leg methods exhibit similar curve shapes with little variation across trajectory lengths. However, Pre-Dog-Leg requires slightly fewer iterations than Dog-Leg for short trajectories, indicating that preprocessing strategies help reduce the number of optimization steps needed.

**Figure 11:**
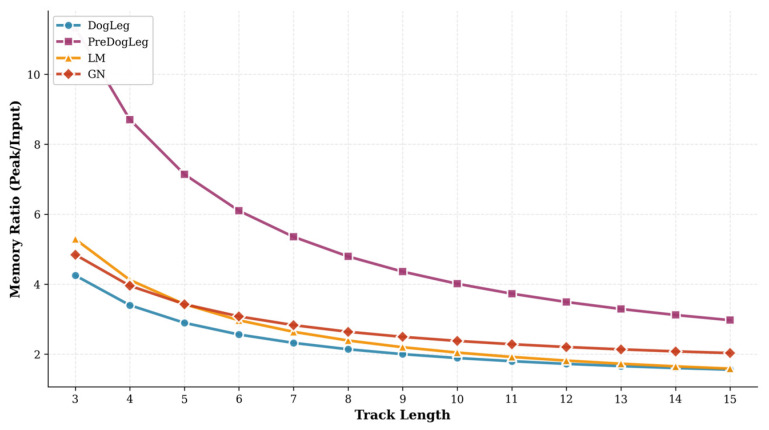
Memory ratio at different track lengths (MH_01).

To validate the actual memory usage of the Pre-Dog-Leg method, we compared the memory efficiency of different approaches. Figure 11 illustrates the variation in peak memory ratio (Memory Ratio) relative to input data size across different trace lengths. It is evident that the Pre-Dog-Leg method consumes significantly more memory than other methods, while the Dog-Leg method (blue) exhibits the lowest memory footprint. The memory ratios for LM and GN are 1.731 and 2.141, respectively, with the memory curve lying between these two values. As summarized in Table 1, the overall average memory ratio for Pre-Dog-Leg is 3.296, which is higher than Dog-Leg’s 1.662. This indicates that Pre-Dog-Leg incurs a greater memory overhead to achieve improved computational speed and solution accuracy.

Finally, regarding convergence accuracy, Table 3 shows that the Pre-Dog-Leg method achieves the highest precision, with an RMSE of only 0.147 m on the MH01 dataset. This represents a reduction of approximately 16% compared to LM and GN, and a reduction of approximately 24% compared to Dog-Leg. This indicates that under the same convergence threshold, the Pre-Dog-Leg method finds a superior solution, resulting in a significant reduction in residual error. In summary, the Pre-Dog-Leg method demonstrates the best overall performance on this dataset: it combines high-speed convergence, comparable to LM, with good stability while significantly improving convergence accuracy. It exhibits clear advantages over GN and Dog-Leg in computational efficiency and result quality, highlighting its value as an improvement. However, it should be noted that the improvements achieved by Pre-Dog-Leg come at the cost of higher memory consumption. This trade-off must be carefully considered in resource-constrained environments.

To further validate the robustness of the Pre-Dog-Leg algorithm in highly dynamic and complex environments, we conducted comparative evaluations on the MH_05 sequence using the Pre-Dog-Leg algorithm, LM algorithm, Gauss–Newton (GN) algorithm, and Dog-Leg confidence region algorithm. The statistical results are presented in the table below.

As shown in Table 4, the computational time distributions of different optimization methods exhibit significant differences. Combined with the statistical results in Table 4, the Pre-Dog-Leg algorithm demonstrates a clear advantage in computational time: its average duration is only 0.116 ms, far below LM’s 0.183 ms, GN’s 0.175 ms, and Dog-Leg’s 0.197 ms. Furthermore, Pre-Dog-Leg’s median computation time of 0.068 ms is close to Dog-Leg’s 0.069 ms and GN’s 0.066 ms, though slightly higher than LM’s 0.053 ms. This indicates that most Pre-Dog-Leg runs also maintain low computational overhead. Notably, the LM algorithm exhibits a long-tail distribution in computation time, with a maximum duration of 36.10 ms. In contrast, Pre-Dog-Leg’s maximum duration is only 19.53 ms, and its standard deviation of 0.651 ms is significantly lower than that of the other methods. This indicates that Pre-Dog-Leg effectively avoids extreme time consumption scenarios, delivering more stable runtime performance. Comprehensive comparisons reveal that Pre-Dog-Leg achieves an average computational time reduction of approximately 37% compared to LM, 34% compared to GN, and 41% compared to Dog-Leg, demonstrating significant acceleration effects. Compared to the MH01 sequence, it offers superior acceleration performance.

**Figure 12:**
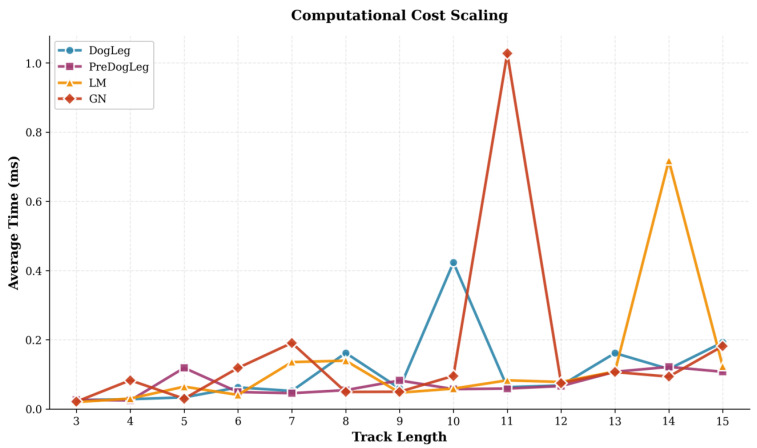
Average convergence time for different trajectory lengths (MH_05).

Next, we examined the impact of trajectory length on algorithm computation time. As shown in Figure 12, the average computation time for each method increases as the trajectory length grows from 3 to 15. For shorter trajectories, the time differences between algorithms are minor, but the gap gradually widens for longer trajectories. The Pre-Dog-Leg algorithm consistently maintained lower average computational time than Dog-Leg across the entire trajectory length range. Even for the longest trajectories, Pre-Dog-Leg exhibited relatively gradual computational time growth and remained more efficient. This demonstrates that Pre-Dog-Leg offers superior scalability with trajectory length, maintaining high computational efficiency as the problem size increases.

**Figure 13:**
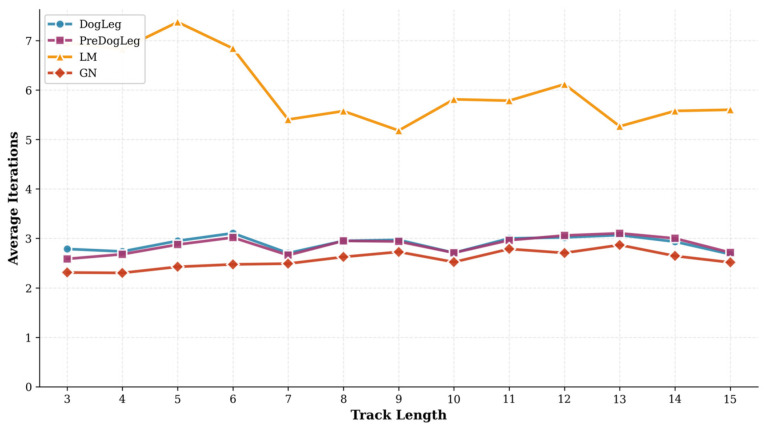
Iteration counts for different trajectory lengths (MH_05).

Furthermore, analyzing the number of iterations for different trajectory lengths in Figure 13 reveals that the average number of iterations varies with trajectory length, indicating differences in the number of iterations required for convergence among the algorithms. The LM algorithm typically converges within fewer iterations. The iteration curve of Pre-Dog-Leg closely resembles that of Dog-Leg, slightly underperforming Dog-Leg at certain trajectory lengths, indicating that preprocessing accelerates convergence speed. Under longer trajectory conditions, Pre-Dog-Leg maintains a low iteration count comparable to Dog-Leg, significantly fewer than the iterations required by GN. Notably, although LM requires the fewest iterations, its per-iteration computational overhead is higher based on runtime results, leading to overall execution time exceeding that of Pre-Dog-Leg. This demonstrates that Pre-Dog-Leg achieves improved overall convergence efficiency through a balanced iteration count and lower per-step computational cost.

**Figure 14:**
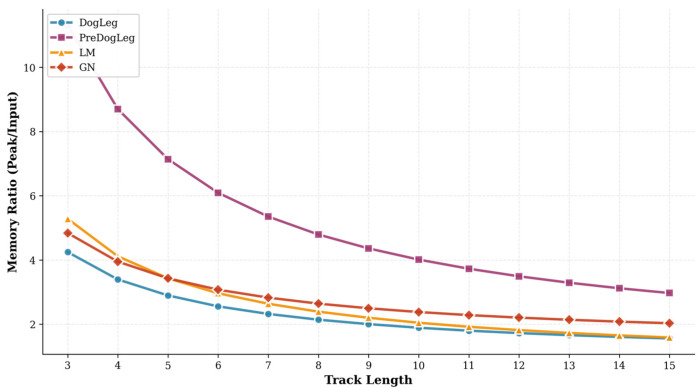
Memory ratio at different track lengths (MH_05).

In the memory overhead comparison shown in Figure 14, each algorithm exhibits distinct differences and trade-offs. The relationship curve between trajectory length and memory consumption reveals that the Pre-Dog-Leg method consistently incurs the highest memory usage. Overall, the Pre-Dog-Leg method incurs higher memory overhead to accelerate computation. As shown in Table 4, Pre-Dog-Leg trades increased memory consumption for faster computation and better convergence performance, reflecting a trade-off between computational time and memory overhead.

The experimental results also compared the convergence accuracy of each method. Pre-Dog-Leg achieved the lowest final convergence error with an RMSE of 0.301, outperforming LM (0.329), GN (0.317), and Dog-Leg (0.353). This demonstrates that Pre-Dog-Leg offers a significant advantage in optimization accuracy while ensuring high-speed convergence.

In summary, on the more dynamically complex MH05 sequence, the Pre-Dog-Leg algorithm demonstrates significantly superior performance compared to other optimization methods. Compared to results on the MH01 sequence, it not only maintains the fastest convergence speed and highest accuracy on MH05 but also further widens the gap between it and the LM, GN, and Dog-Leg algorithms, showcasing superior optimization capabilities in complex dynamic scenarios. However, it should be noted that this advantage comes at the cost of higher memory consumption, requiring careful consideration in resource-constrained environments.

### 4.3. Pre-Dog-Leg Sensitivity Test Experiment

This experiment conducted a sensitivity analysis on the preconditioner threshold parameter in the Pre-Dog-Leg algorithm. The preconditioner threshold determines when the combined preconditioner (SPAI-Jacobi) is activated, specifically when the condition number of the Hessian matrix exceeds this value. We selected three representative sequences: MH_01, MH_02, and MH_05 from the EuRoC dataset, representing varying levels of difficulty. Comparative tests were conducted with threshold values set at 500, 1000, 1500, 2000, and 10,000.

Table 5 summarizes the average convergence time per frame (seconds) and average trajectory error (ATE, meters) for each sequence when using thresholds of 500, 1000, 1500, 2000, and 10,000. It can be observed that for simple scenes (MH_01 and MH_02), the trajectory positioning accuracy of the algorithm varies across different thresholds, with only minor changes in convergence time. However, for the challenging MH_05 sequence, the threshold setting has a more significant impact on performance.

**Figure 15:**
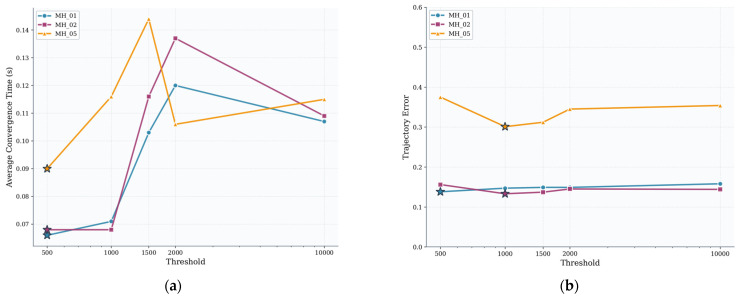
Comparison of average convergence time and trajectory error. (**a**) Average convergence rate; (**b**) trajectory error comparison.

Figure 15a illustrates the trend of the average convergence time for the Pre-Dog-Leg algorithm as the precondition threshold varies (500, 1000, 1500, 2000, and 10,000). Figure 15b shows the trajectory error (ATE) as a function of the threshold. It can be observed that in the MH_01 and MH_02 scenarios, different thresholds have a minor impact on the average convergence time, while ATE fluctuates slightly. In contrast, in the complex MH_05 scenario, a threshold of 1000 achieves a balance between convergence speed and accuracy, minimizing ATE and delivering the overall optimal performance. Conversely, when the threshold increases to 10,000, MH_05’s ATE surges dramatically, indicating that excessively high thresholds severely compromise accuracy. A comprehensive comparison of Figure 15a,b reveals that the threshold = 1000 condition achieves balanced performance across all three scenarios, demonstrating a distinct advantage, particularly in the complex MH_05 scenario.

### 4.4. Pre-Dog-Leg Localization Accuracy Experiment

We selected the MH Factory dataset to validate the robustness and accuracy of our algorithm under various environments. To better demonstrate this, we conducted comparative experiments with RVIO2 and VINS-mono. We disabled VINS-mono’s loop detection feature and OpenVINS’ [39] internal parameter optimization function. Using identical EuRoC configurations, we evaluated our algorithm’s absolute trajectory error (ATE) against RVIO2 and VINS-mono as benchmarks.

Table 6 presents the quantitative comparison results of the absolute pose error (ATE) between the algorithm proposed in this paper and RVIO2 and VINS-mono. These results are based on the MH series sequences of the EuRoC dataset. The evaluation metrics include maximum error (Max), mean error (Mean), median error (Median), minimum error (Min), root mean square error (RMSE), sum of squared errors (SSEs), and standard deviation (std), all in meters. Bolded values indicate the best performance of our algorithm for that metric.

As shown in Table 6, our algorithm achieves a significant reduction in RMSE across three sequences. The RMSE distribution of our method remains below 0.15, whereas RVIO2 exhibits an RMSE of 0.176 m, with VINS-mono at 0.161 m, and OpenVINS at 0.205 m. Our algorithm achieves an RMSE of only 0.147 m, representing a reduction of approximately 16.48% compared to RVIO2. It reduces the RMSE by approximately 8.70% compared to VINS-mono and by about 28.29% compared to OpenVINS. For the MH_02 sequence, RVIO2 has an RMSE of 0.138 m, VINS-mono has 0.168 m, and OpenVINS has 0.291 m. Our method reduces this to 0.133 m, representing a reduction of approximately 20.83% compared to VINS-mono. For the MH_02 sequence, the RMSE was 0.138 m for VINS-mono and 0.168 m for our method, which was reduced to 0.133 m—a decrease of approximately 20.83% compared to VINS-mono.

**Figure 16:**
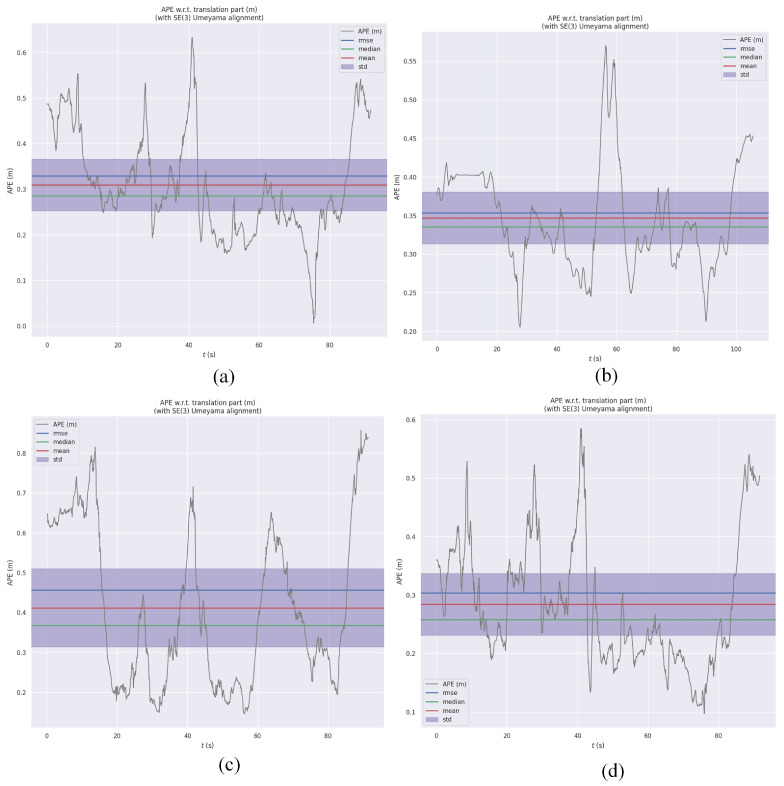
Absolute trajectory error variation diagram: (**a**) RVIO2 on the MH_05 sequence; (**b**) VINS-mono on the MH_05 sequence; (**c**) OpenVINS on the MH_05 sequence; (**d**) Our algorithm on the MH_05 sequence.

**Figure 17:**
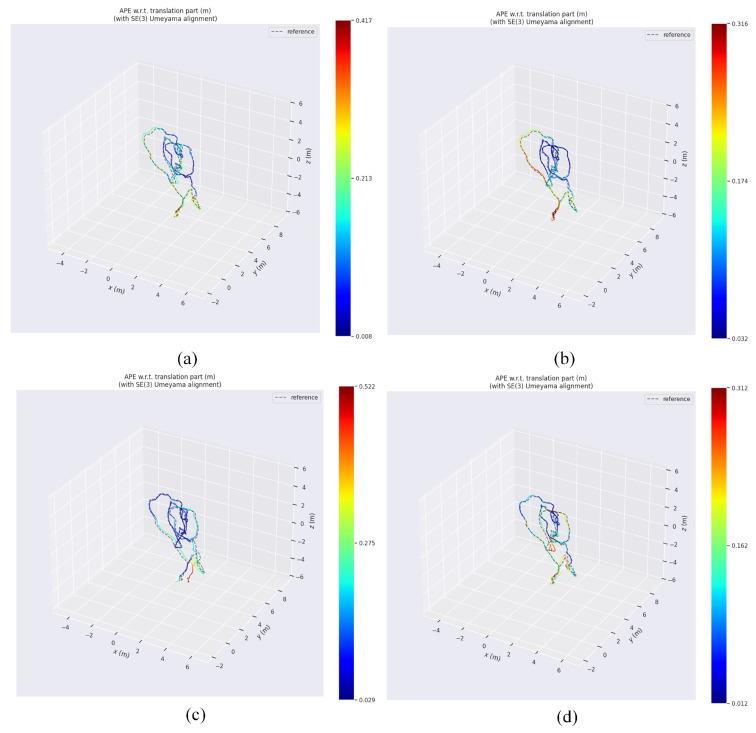
Trajectory error heat map. (**a**) RVIO2 on the MH_01 sequence; (**b**) VINS-mono on the MH_01 sequence; (**c**) OpenVINS on the MH_01 sequence; (**d**) Our algorithm on the MH_01 sequence.

Figure 16a–d show the RMSE of RVIO2, VINS-mono, OpenVINS, and our algorithm on the MH_05 sequence, respectively. Quantitative analysis reveals that RVIO2 has an RMSE of 0.329 m, VINS-mono has 0.353 m, OpenVINS has 0.456 m, and our algorithm has 0.301 m, demonstrating our algorithm’s superior robustness during high-dynamic, long-duration operations. Comparing the maximum error, mean error, and median error in Table 3 reveals that our algorithm demonstrates a clear advantage in the stability of error distribution. Furthermore, we conducted a reliability analysis of the algorithm from the perspective of maximum and minimum errors, visualizing the specific results in the figure below:

Figure 17a–d clearly shows that on the MH_01 sequence, RVIO2 exhibits a maximum error of 0.417 m, VINS-mono has a maximum error of 0.316 m, OpenVINS reaches 0.522 m, while our method achieves only 0.312 m—representing a reduction of approximately 25.2% compared to RVIO2. On the MH_02 sequence, RVIO2 exhibits a maximum error of 0.324 m, VINS-mono shows a maximum error of 0.517 m, while our method achieves only 0.294 m. This represents a reduction of approximately 43.13% compared to VINS-mono and about 9.26% compared to RVIO2, indicating that the algorithm effectively suppresses the generation of outliers. On the MH_05 sequence, RVIO2 exhibited a maximum error of 0.633 m, VINS-mono reached 0.570 m, and OpenVINS recorded 0.857 m, while our method achieved 0.585 m—a reduction of approximately 7.58% compared to RVIO2. Simultaneously, the reduction in standard deviation further confirms the stability of the estimation results. The minimum error metric reflects the algorithm’s optimal performance under ideal conditions. Except for the MH_03 sequence, the minimum error remained at a low level across all other sequences. Notably, on the MH_02 sequence, the error decreased from 0.020 m to an extremely low 0.002 m, validating the algorithm’s high-precision potential under favorable observation conditions.

In summary, the experimental results show that the improved Dog-Leg algorithm based on the preconditioner proposed in this paper can effectively improve the positioning accuracy and stability of visual inertial navigation. It is worth noting that in the MH_05 high-dynamic long motion sequence, the algorithm proposed in this paper has higher adaptability and robustness, demonstrating higher accuracy. As for MH_03, which is a dataset with dense pipe texture feature repetitions, future research will add verification steps to this algorithm to reduce the probability of feature mismatches.

## 5. Conclusions and Future Work

This paper addresses the challenge of feature point optimization in visual-inertial SLAM systems by proposing the Pre-Dog-Leg method based on a preconditioner. This approach effectively resolves the slow convergence and non-convergence issues encountered by the traditional Dog-Leg algorithm when handling ill-posed problems. Experimental results demonstrate that this method reduces the average condition number of the Hessian matrix from 2.7 × 10^3^ to 3.4 × 10^2^, achieving a 7.9-fold improvement factor and significantly enhancing numerical stability. In terms of convergence performance, the overall convergence time improved by approximately 50.81% compared to the original Dog-Leg algorithm. Notably, on the MH_05 high-dynamic sequence, Pre-Dog-Leg averaged only 0.116 s—37% faster than the LM algorithm and 41% faster than Dog-Leg. Positioning accuracy demonstrated exceptional performance: the MH_01 sequence achieved an RMSE of 0.147 m, representing a 16.48% reduction compared to RVIO2, an 8.70% improvement over VINS-mono, and a 28.29% decrease relative to OpenVINS; The MH_02 sequence achieved an RMSE of 0.133 m, representing a 20.83% reduction compared to VINS-mono and a substantial 54.30% improvement over OpenVINS. On the complex MH_05 high-dynamic sequence, the RMSE was 0.301 m, 8.51% lower than RVIO2, 14.73% lower than VINS-mono, and 34.0% lower than OpenVINS. Although the memory utilization ratio of this method is approximately 3.3, higher than Dog-Leg’s 1.66, it achieves significant computational speedup and accuracy improvement at roughly double the memory cost, which is acceptable for practical applications. Sensitivity analysis further validated the algorithm’s robustness. When the preconditioner threshold was set to 1000, the algorithm demonstrated optimal balanced performance across various scenarios, providing a more efficient and stable optimization solution for high-dynamic, long-duration SLAM systems.

In SLAM problems, the numerical solution of optimization algorithms directly determines the system’s real-time performance and accuracy. Future research will focus on advancing the deep integration and application expansion of the Pre-Dog-Leg algorithm in SLAM systems: On one hand, this will involve extending Pre-Dog-Leg to the backend pose graph optimization module to address multi-scale parameter coupling challenges in large-scale map optimization; on the other hand, deploying the Pre-Dog-Leg algorithm in multi-modal fusion systems (e.g., LiDAR-vision-inertial) will involve designing hybrid preconditioner strategies that fully account for heterogeneous sensor measurement characteristics, significantly enhancing numerical stability. These technological innovations will lay a solid foundation for constructing efficient and robust next-generation SLAM systems, demonstrating significant practical value, especially in complex dynamic environments and resource-constrained platforms.

## Figures and Tables

**Table 1:** Key research findings summary.

Aspect	The Global State Is Unobservable				Feature Point Information Is Not Observable	
	FEJ	PC-SRIF	RVIO2	SRIF	[30]	[32]
Issue	Information matrix null space	Single-precision Cholesky stability issues	Global frame changes lead to instability	Sliding-window BA Hessian ill-conditioned	Pure rotation (no parallax) + feature mismatches	Pure rotation/static motion yields Hessian singularity
Target Block	Global State and Linearization	Sliding Window Optimization	State Estimation Framework	Nonlinear Solve	Deep learning + priors	Front-end feature matching
Limitations	No extra observations; unobservable issues persist	Depends on initial guess; poor initialization causes linearization errors	Inherent unobservability persists	Inherent unobservability persists	Relies on deep learning for scale estimation	Relies on IMU for scale estimation

**Table 2:** Euroc sequence list.

Dataset	Distance (m)	Average Linear Velocity (m/s)	Average Angular Velocity (Rad/s)	Duration (s)	Difficulty/Characteristics
MH_01	80.6	0.44	182	0.22	good texture, bright
MH_02	73.5	0.49	150	0.21	good texture, bright
MH_03	130.9	0.99	132	0.29	fast motion, bright
MH_05	97.6	0.88	111	0.21	fast motion, dark

**Table 3:** MH_01 sequence performance comparison (bold values denote optimal performance).

Method	Convergence Rate (ms)					Memory Ratio	RMSE (m)
	Max	Min	std	Median	Mean		
LM	9.422	0.009	0.266	0.050	0.071	1.7309	0.176
GN	28.370	0.009	0.633	0.063	0.109	2.1405	0.175
Dog-Leg	25.369	0.014	0.515	0.066	0.093	**1.6619**	0.193
Pre-Dog-Leg	10.336	**0.013**	**0.251**	0.064	**0.071**	3.2962	**0.147**

**Table 4:** MH_05 sequence performance comparison (bold values denote optimal performance).

Method	Convergence Rate (ms)					Memory Ratio	RMSE
	Max	Min	std	Median	Mean		
LM	36.10	0.010	1.241	0.053	0.183	1.836	0.329
GN	25.13	0.012	0.992	0.066	0.175	2.145	0.317
Dog-Leg	30.67	0.017	1.120	0.069	0.197	**1.667**	0.353
Pre-Dog-Leg	**19.53**	0.014	**0.651**	0.068	**0.116**	3.316	**0.301**

**Table 5:** ATE and average convergence time at different thresholds (bold values denote optimal performance).

Threshold	MH_01		MH_02		MH_05	
	Avg. Convergence Time (ms)	ATE (m)	Avg. Convergence Time (ms)	ATE (m)	Convergence Time (ms)	ATE (m)
500	**0.066**	0.138	0.068	0.156	0.090	0.375
1000	0.071	0.147	**0.068**	**0.133**	0.116	**0.301**
1500	0.103	0.149	0.116	0.137	0.144	0.312
2000	0.120	0.149	0.137	0.145	0.106	0.345
10,000	0.107	0.158	0.109	0.144	**0.115**	0.354

**Table 6:** Comparison of absolute trajectory error (bold values denote optimal performance).

Method	Dates	Max	Mean	Median	Min	RMSE	SSE	std
RVIO2	MH_01	0.417	0.159	0.151	0.008	0.176	86.27	0.084
	MH_02	0.324	0.122	0.104	0.020	0.138	42.90	0.064
	MH_03	0.597	0.174	0.162	0.014	0.199	93.27	0.097
	MH_05	0.638	0.310	0.286	0.007	0.329	198.7	0.113
Vins-mono	MH_01	0.316	0.143	0.118	0.032	0.161	36.01	0.075
	MH_02	0.517	0.127	0.086	0.033	0.168	42.08	0.110
	MH_03	0.420	0.162	0.157	0.010	0.182	43.41	0.082
	MH_05	0.570	0.347	0.335	0.205	0.353	132.2	0.067
OpenVINS	MH_01	0.522	0.175	0.146	0.028	0.205	116.07	0.107
	MH_02	0.693	0.247	0.191	0.013	0.291	190.17	0.154
	MH_03	0.635	0.259	0.244	0.045	0.284	184.73	0.116
	MH_05	0.857	0.412	0.368	0.146	0.456	381.05	0.196
Pre-Dog-Leg	MH_01	**0.312**	**0.132**	0.124	0.011	**0.147**	59.98	**0.065**
	MH_02	**0.294**	**0.119**	0.111	**0.002**	**0.133**	**40.29**	**0.061**
	MH_03	0.581	0.192	**0.157**	0.046	0.220	113.33	0.105
	MH_05	0.585	**0.028**	**0.026**	0.010	**0.301**	168.8	0.106

## Data Availability

Data related to the current study are available from the corresponding author upon reasonable request. The codes used during the study are available from the corresponding author upon request.
